# Cortisol in Oral and Maxillofacial Surgery: A Double-Edged Sword

**DOI:** 10.1155/2021/7642875

**Published:** 2021-09-02

**Authors:** Muslat A Bin Rubaia'an, Muath Khaled Alotaibi, Naif Mutlaq Alotaibi, Nasser Raqe Alqhtani

**Affiliations:** ^1^College of Dentistry, Riyadh Elm University, Riyadh, Saudi Arabia; ^2^Saudi Board for Oral and Maxillofacial Surgery, Presidency State of Security, Riyadh, Saudi Arabia; ^3^Saudi Board for Oral and Maxillofacial Surgery, Saudi Armed Forces, Ministry of Defense, Riyadh, Saudi Arabia; ^4^Department of Oral and Maxillofacial Surgery and Diagnostic Science, College of Dentistry, Prince Sattam Bin Abdulaziz University, Alkharj, Saudi Arabia

## Abstract

Cortisol is a hormone that is naturally produced by the zona fasciculata of the cortex in the adrenal gland. One of its main functions is to decrease inflammation, particularly in areas where an inflammatory response is not necessary. In the field of oral and maxillofacial surgery, cortisol is used to improve the outcomes of surgical procedures and to make the postoperative period more comfortable for the patient. However, cortisol is considered a double-edged sword because its use is associated with both benefits and adverse effects. It is imperative to use cortisol following an accurate diagnosis, in addition to clarity regarding the desired surgical procedure for treating the acute or chronic condition affecting the patient. When used with caution, cortisol can serve as a valuable agent for reducing the postoperative inflammatory response in patients undergoing moderate as well as moderately severe surgical procedures.

## 1. Introduction

Cortisol is a steroid hormone that the body naturally secretes in minute quantities. This natural steroid plays a crucial role in maintaining homeostasis in the body, and its synthetic analogs are extensively used in the treatment of various medical conditions [1]. Cortisol is mainly used to reduce inflammatory reactions following surgical procedures. The potential of cortisol for reducing postoperative edema was first explored by Hooley and Hohl in 1974; they observed that the use of topical steroids following surgical procedures on the lips and oral commissures significantly reduced ulcers and excoriation and subsequently reduced postoperative tissue retraction [2]. Cortisol is a very potent drug against inflammatory responses and is thus frequently prescribed for various conditions [1]. Careful consideration is required before initiating the use of cortisol and its synthetic analogs in patients undergoing oral and maxillofacial surgery. The most frequently prescribed steroids include dexamethasone, methylprednisolone, dexamethasone acetate, dexamethasone sodium phosphate, methylprednisolone acetate, and methylprednisolone sodium succinate, which can be administered orally, intramuscularly, intravenously, or intramuscularly [2]. The use of cortisol and its synthetic analogs that have a long-acting action is generally preferred. Dexamethasone is considered to be 25–30 times more potent than cortisol and is usually prescribed by clinicians for administration via different routes, including oral, parenteral, and topical [3]. On the other hand, the potential role of cortisol in clinical practice remains debatable. The use of cortisol is generally considered only in patients undergoing severe surgical procedures. However, it is suggested to be a valuable agent in patients undergoing both moderate and moderately severe surgical procedures [4]. Given its critical role in reducing postoperative inflammatory response in the body, the use of cortisol can lead to a substantial decrease in morbidities associated with oral and maxillofacial surgery. The current review provides an overview of the role of cortisol and emphasizes its uses, contraindications, and side effects when administered to patients undergoing oral and maxillofacial surgery.

### 1.1. Physiology of Cortisol

**Figure 1:**
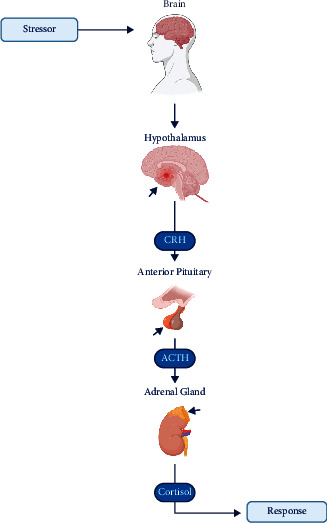
The cortisol pathway [6]. CRH: corticotropin-releasing hormone and ACTH: adrenocorticotropic hormone (created with BioRender.com).

The adrenal cortex secretes three types of hormones: glucocorticoids, androgens, and mineralocorticoids. Within the adrenal cortex, the zona fasciculata secretes the glucocorticoids cortisol and corticosterone, in addition to secreting small quantities of adrenal androgens and estrogens [5] (Figure 1). These are also central to the body's response to stress and the physiological response of the vascular system to tissue damage [7, 8]. A total of 24–30 mg/day of cortisol is secreted by the adrenal gland in normal adults [9]. However, during periods of stress, this secretion level can spike up to 300 mg/day [3]. Diurnal variations; the response of individuals to stress; and the effectiveness of control mechanisms involving the hypothalamus, pituitary gland, and adrenal glands play a central role in regulating the daily secretion level of cortisol. When high doses of corticosteroids equivalent to >30 mg of cortisol are administered for two weeks, these regulatory mechanisms may be altered and may return to normal only over a period of up to one year. Meanwhile, the body's capacity to react to stress can recover in just 15–20 days [9, 10]. A total of 10–20 mg/day of cortisol is secreted, with almost 50% of this being secreted in the early hours of the day [3].

### 1.2. Mechanism of Action of Cortisol

During and after any surgical procedure, classical signs of inflammation, including rubor, calore, dolore, tumor, and functio laesa, can be observed; these terms refer to redness, elevated temperature, increased pain level, tissue enlargement due to fluid extravasation, and loss of function, respectively [11]. An appropriate inflammatory response is critical for an effective healing process in response to tissue damage. Cortisol plays a central role in reducing the signs and symptoms of inflammation by inhibiting the enzyme phospholipase A2, which converts phospholipids into arachidonic acid. This in turn blocks the formation of other products, such as prostaglandins, leukotrienes, and thromboxane A2. Therefore, cortisol is critical for inhibiting the formation of inflammatory mediators, which are mainly responsible for the adverse effects associated with the inflammatory response in the body [9]. Cortisol also makes the lysosomal membranes more stable, thereby reducing the release of lysozymes, which propagate an inflammatory response at the site of injury. Furthermore, cortisol reduces capillary permeability, which in turn reduces inflammatory fluid extravasation and diapedesis.

### 1.3. Contraindications

The use of cortisol is contraindicated in patients with bacterial infection of primary origin, hypersensitivity reactions, gastric ulcers, elevated blood glucose and blood pressure levels, osteoporosis, herpes simplex infection, epilepsy, mental illnesses (including psychosis), congestive heart failure, or renal failure [1] (Table 1). Evidence for the use of topical cortisol in pregnant women is not unequivocal. Statistically significant associations have been reported between the use of topical cortisol and its related drugs and congenital anomalies, such as low birth weight and orofacial clefts [12]. The use of topical cortisol is therefore not recommended in pregnant women.

### 1.4. Side Effects and Their Management

**Figure 2:**
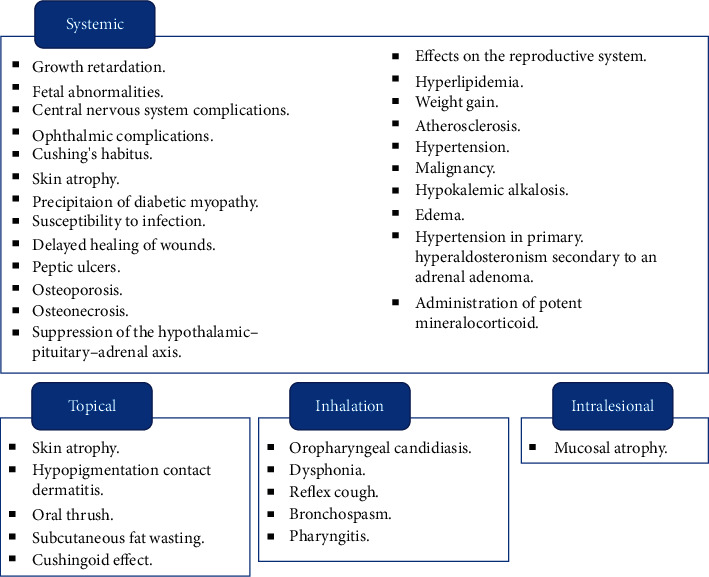
Side effects of cortisol according to the route of administration [12, 15–17].

The side effects associated with the use of cortisol have been well documented in the literature and depend on the dosage administered and the duration of therapy [13, 14]. Moreover, the route of administration has a major impact on the side effects experienced by the patient (Figure 2) [12, 15–17]. The general side effects include weight gain, growth retardation, adrenal insufficiency, an increase in the tendency to acquire infections, weakening of the bones (leading to osteoporosis and fracture), osteonecrosis, gastric ulcers, alterations in sugar control and glucose metabolism, myopathy, glaucoma, cataract, and insomnia. In addition, steroid withdrawal syndrome may be experienced by some patients [18]. This syndrome is defined as an objective syndrome that resembles true adrenal insufficiency and is characterized by fever, anorexia, nausea, lethargy, malaise, arthralgia, desquamation of the skin, weakness, and weight loss that occur (with highly variable grades) in patients undergoing steroid withdrawal due to biochemical evidence of suppressed hypothalamus-pituitary-adrenal (HPA) system integrity [18]. Generally, a regimen period less than 7–14 days does not develop HPA suppression. Hence, corticosteroids can be suspended safely without tapering [19]. The suppression of HPA correlates with the long-term administration of an equivalent dose of 40 mg/day of cortisol or greater (Table 2) [14,20]. Commonly used steroids are different in respect to their anti-inflammatory potency and duration of action, and therefore, their responsible dosages of HPA suppression (Table 3). The clinical signs and symptoms of adrenal insufficiency manifest when the blood levels of cortisol are constantly high for more than five days or when cortisol is consistently administered to the patient for 14 days. It is therefore recommended that the use of cortisol be tapered and not stopped abruptly [24]. The purpose of this gradual tapering is to allow the pituitary and adrenal gland to recover their normal secretions, which is equivalent to 5–7.5 mg/day of prednisone (equivalent to 24–30 mg/day of cortisol), without exacerbating the state of the underlying disease [14, 25]. As a general role and depending on dosage, a decreasing dosage by the equivalent of 2.5–5 mg/day of prednisone every 3–7 days ending with a dosage of 5 mg/day of prednisone should be prescribed [25]. The use of agents such as probiotics can have a major impact on reducing the side effects associated with cortisol therapy. In particular, probiotics can reduce the impact of candidiasis in patients receiving cortisol. One of the major mechanisms of action is the inhibition of pathogenic gut microbes. Probiotics increase mucus production in the gut, thereby enhancing the activity of the gut mucosal barrier and improving gut mucosal integrity. This results in the synthesis of short-chain fatty acids in the gut mucosa, increases the production of secretory immunoglobulin, and reduces the expression of tumor necrosis factor. These effects of probiotics are attributed to the induction of interleukin-10 [26].

#### 1.4.1. Adrenal Insufficiency

Most patients with adrenal insufficiency do not require supplementation with glucocorticoids for routine dental treatments. This is because routine dental procedures do not lead to a spike in cortisol levels in the body in comparison with oral and maxillofacial surgical procedures. The administration of local anesthetic solutions effectively inhibits the neural pathways triggered by stress, which are central for the secretion of adrenocorticotropic hormones [27]. However, a corticosteroids supplementation protocol is recommended perioperatively for patients undergoing different stressful situations (Table 4) [1].

## 2. Uses of Cortisol in Oral Surgery

The use of cortisol and its related drugs is crucial in patients undergoing oral and maxillofacial surgery. These agents reduce the extravasation of fluids from the intravascular compartment to the extravascular compartment, thereby limiting postoperative edema. Cortisol has been successfully used in the treatment of chronic orofacial pain, acute trigeminal nerve injuries, allergic manifestations in the orofacial region, and traumatic facial nerve paralysis, in addition to being used in orthognathic surgery [12, 28, 29]. Although corticosteroids are most effective during the first 24 hours after surgery, their effect can be observed for up to three postoperative days. Many authors have reported a statistically significant reduction in pain after the postoperative administration of 40 mg of methylprednisolone, 25 mg of prednisolone, or 0.5 mg of betamethasone [30, 31]. One study reported that the oral administration of 8 mg of dexamethasone before surgery can significantly reduce pain and inflammation [32]. Other drugs can also induce profound analgesia among patients. For example, steroids can decrease the levels of beta-endorphins in the body, thereby decreasing the patients' response to pain. Moreover, given their euphoric effect, they can alter the moods of patients, which can aid in postoperative recovery. It should be noted that as a therapeutic agent, cortisol should be administered in doses that are above the physiological quantities that are normally produced in the body. Among the frequently prescribed steroids, dexamethasone is preferred over methylprednisolone in patients undergoing oral and maxillofacial surgery because its effects last for longer periods of time [33]. There are diverse opinions on performing oral and maxillofacial surgical procedures in patients who have stopped using steroids in recent times. A waiting period of 15 days is preferred for the restoration of normal functioning and secretion of adrenal glands prior to performing elective oral and maxillofacial surgical procedures. Patients who are being administered either 30 mg of cortisol or 5 mg of prednisone or less may be eligible to undergo surgical procedures without this waiting period [34].

### 2.1. Surgical Extractions

The surgical extraction of mandibular third molars is considered the most common oral surgical procedure [35]. Patients experience a range of adverse signs and symptoms following extraction, including pain, facial edema, and trismus due to masticatory muscles inflammation [36]. In such patients, corticosteroids can exert a crucial anti-inflammatory effect by reducing fluid transudation and edema formation, decreasing cell exudates, inhibiting vascular dilatation, and reducing fibrin deposition around the inflamed area. The mechanisms responsible for these effects include the restriction of leukocyte chemotaxis to the inflammatory focus, inhibition of fibroblast and endothelial cell function, and suppression of the production of numerous chemical mediators of inflammation. Various studies have suggested the anti-inflammatory role of cortisol in patients undergoing oral surgical procedures [30–32]. The degree of facial swelling has been found to be 42% lesser in patients administered methylprednisolone 48 hours after surgery [31, 37, 38]. The oral administration of 8 mg of dexamethasone either preoperatively or postoperatively has been shown to reduce postoperative complications [35]. It has been found that the duration of action of this dosage is 66 hours, which can be considered sufficient since it covers the first three days of maximum facial swelling's inflammatory process [39]. Moreover, cortisol has been administered in combination with other drugs to reduce postoperative complications. Favorable effects of such combinations have been observed in terms of reducing the level of difficulty in opening the mouth and the degree of pain and swelling in patients [37, 38, 40, 41]. Given its analgesic and anti-inflammatory effects, a combination of diclofenac and prednisolone has been found to be highly effective in reducing postoperative pain and swelling [38].

### 2.2. Temporomandibular Disorders (TMDs)

TMD is a collective term for a group of musculoskeletal and neuromuscular disorders that produce clinical signs and symptoms involving the temporomandibular joints (TMJs), together with the muscles of mastication and associated structures [42]. TMDs are the third most prevalent disorders causing pain worldwide [43]. Difficulty in opening the mouth, pain, tenderness in the muscles of mastication, and deviation in jaw movements are frequent manifestations of TMDs. Cortisol has been used in the management of patients with signs and symptoms of TMDs or TMJ arthritis. It has also been used to diagnose TMDs [44, 45]. The adopted doses of cortisol and its analogs for the management of TMDs are as follows: 20–240 mg/day of cortisol, 5–60 mg/day of prednisone, 5–60 mg/day of prednisolone, 0.6–7.2 mg/day of betamethasone, and 0.75–9 mg/day of dexamethasone. Researchers have also reported favorable results following the intra-articular injection of cortisol into the TMJ. Moreover, they have reported that TMJ functions revert back to normal after the administration of cortisol [46–48].

### 2.3. Mucocele

Mucocele is the most common minor salivary gland lesion [49]. The nonsurgical procedure is preferred over the surgical procedure for treating mucoceles. The surgical procedure has numerous disadvantages, such as lip disfigurement and damage to adjacent vital structures. A 1 mL intralesional injection of 4 mg/mL of betamethasone is reported to be effective in treating mucoceles. The solution should be injected adjacent to the periphery and into the base of the lesion [50].

### 2.4. Oral Submucous Fibrosis

Cortisol and its synthetic analogs have been topically used in patients with oral submucous fibrosis, particularly in patients with ulcers and mucosal pain. Symptomatic relief is achieved because of the anti-inflammatory properties of cortisol [51, 52]. The use of a topical triamcinolone ointment thrice a day is preferred [53].

### 2.5. Oral Lichen Planus

The preferred drug for the treatment of oral lichen planus is 0.05% clobetasol propionate; it is delivered via a gingival tray. This ointment should be applied twice a day in a period of 4 months and then tapered into once a day for 2 more months [54]. It is advisable to administer 100,000 IU/mL of nystatin via Orabase (Colgate-Palmolive, New York, NY, USA) along with this drug to prevent candidiasis [17].

### 2.6. Erythema Multiforme

During the initial stages of treatment of erythema multiforme, 0.5–1.0 mg/kg/day of prednisone may be administered systemically. Tapering this dose over 7–10 days is suggested. Alternatively, 1 mg/kg/day of pulse methylprednisolone may be administered for three days. The use of oral prednisone is not as effective as that of intravenous methylprednisolone administered as a pulsed dose of 20–30 mg/kg of daily infusions for three consecutive days. A maximum of 500 mg can be administered over a period of three to four hours. The beneficial effects of the treatment are observed earlier with intravenous administration than with oral administration. This leads to substantial improvements in the patient's condition and halts the progression of the cutaneous lesion [55].

### 2.7. Pemphigus Vulgaris

Pulse therapy is the preferred modality for treating pemphigus vulgaris. It involves the use of 500–1,000 mg of prednisolone or 100–200 mg of dexamethasone. It can be observed that the dose for each pulse is not standardized. This aims in achieving a faster response without the need of long-term regimen. The combined use of immunosuppressive agents is preferred for obtaining better results [56].

### 2.8. Bullous Pemphigoid

Cortisol and its related drugs are used in moderate doses for the effective management of bullous and mucous membrane pemphigoid. A mild regimen consisting of topically applying 20 g of 0.005% clobetasol propionate if the patient's weight is less than 45 kg, or 30 g if it is more than 45 kg has been shown to be effective [57]. This dosage should be applied until 15 days of controlling the disease; then, the doses are tapered to 20 or 30 g every other day for one month, 20–30 g twice weekly for another one month, and 20–30 g once weekly for the last one month, respectively.

### 2.9. Bell's Palsy

In patients with Bell's palsy, 60 mg/day of prednisolone is administered during the first five days of treatment, following which the dosage is tapered over the next six days with a reduction of 10 mg/day ending with an 11-day regimen [58]. It has to be borne in mind that the treatment should be initiated during the first 72 hours of symptom onset.

### 2.10. Central Giant-Cell Granuloma

The nonsurgical management of central giant-cell granuloma includes the administration of cortisol and its related drugs via an intralesional injection. To suppress the angiogenic properties of the granuloma, a concurrent topical application of triamcinolone acetonide is preferred [59]. The use of a 10 mg/mL injection of triamcinolone acetonide and 0.5% lignocaine in equal parts once a week for a total of five weeks has been reported to be effective over a 3-year follow-up period [60].

### 2.11. Postherpetic Neuralgia

The systemic administration of steroids is preferred for pain management in patients with postherpetic neuralgia [56]. The use of 40 mg/day of prednisone for 10 days is safe and effective in reducing the incidence of postherpetic neuralgia [3]. This dose should be gradually tapered in the following 21 days.

### 2.12. Melkersson–Rosenthal Syndrome

Cortisol and its related drugs are preferred for their anti-inflammatory effects and can help reduce edema and swelling in patients with Melkersson–Rosenthal syndrome. The preferred drug for such patients is prednisolone at a dose of 1–1.5 mg/kg/day. Based on the patient's response to the drug and the severity of the lesion, the dose of prednisolone can be tapered over a period of 21–42 days [56].

## 3. Emergency Scenarios

### 3.1. Adrenal Crisis

Acute adrenal crisis is a medical emergency characterized by cortisol insufficiency. The symptoms of cortisol insufficiency include abdominal pain, lethargy, nausea, vomiting, dehydration, and hypotension. Hyperkalemia, hyponatremia, hypoglycemia, uremia, and acidosis may also be observed in such patients. Administering cortisol externally can increase adrenal gland suppression and atrophy. This may lead to an inability of the body to react to stress and can further worsen an adrenal crisis. To manage an adrenal crisis, fluids such as 5% dextrose in normal saline or 20–25 mg of cortisol per 24 hours for primary adrenal insufficiency and 15–20 mg of cortisol per 24 hours for secondary adrenal insufficiency should be administered, with cortisol being intravenously administered in the beginning. Improvements in the patient's condition can indicate the need to reduce the dose further. The oral route of administration should be preferred if the patient's condition improves. Moreover, the medication dose can be gradually tapered in such cases, with 20 mg being administered in the morning and 10 mg at midday or night. A total dose of 20 mg may be required in some patients and can be administered entirely in the morning or divided into smaller doses to be provided in the morning and at midday or night. One of the important principles of management is the need to treat the underlying cause of the adrenal crisis. The administration of a mineralocorticoid may not be required [61].

### 3.2. Anaphylactic Shock

An anaphylactic shock is one of the most important events in emergency medicine. If the onset of anaphylaxis occurs four to six hours after exposure to the allergen, the administration of steroids may not be useful in treating acute anaphylaxis. There is a dearth of evidence regarding the amount of steroids that can be used to effectively manage anaphylaxis in patients on antihistamines. In patients with airway obstruction and bronchospasm, 1–50 mg/kg prednisone can be administered orally or 1.5–3 mg/kg cortisol can be administered intravenously. This recommendation is based on the management of patients with asthma. Recurrent idiopathic anaphylaxis can be managed by administering steroids [62].

## 4. Conclusion

Cortisol has been used for the management of a wide variety of conditions related to the head and neck. Moreover, it plays a key role in the management of postoperative complications, such as swelling and pain, after oral and maxillofacial surgery. Therefore, cortisol has been rightly labeled a life-protecting drug, while aldosterone has been labeled a life-saving drug. It should be noted that cortisol and its related drugs have side effects that can severely harm the patient and, in some instances, be fatal. Hence, the use of cortisol as a therapeutic agent is considered a double-edged sword. It is important to critically assess and analyze the risk-benefit profile of the clinical application of cortisol on a case-to-case basis.

## Figures and Tables

**Table 1:** Contraindications of cortisol use according to the route of administration [1].

Mode of delivery	Contraindications
Systemic	Psychiatric disorders, glaucoma, cataract, underactive thyroid, heart diseases, tuberculosis, mycobacterial diseases, active peptic ulcer, pregnancy, diabetes, osteoporosis, infections, herpes simplex infection, varicella-zoster infection, and immune deficiency diseases
Topical	Primary bacterial infections, such as impetigo, furuncles, erysipelas, carbuncles, cellulitis, and lymphangitis, and hypersensitivity to any component of the preparation
Parenteral	Hypersensitivity to corticosteroids, active tuberculosis, and infections

**Table 2:** Equivalent doses of numerous corticosteroids.

40 mg of cortisol is equivalent to	10 mg of prednisone
	10 mg of prednisolone
	8 mg of methylprednisolone
	8 mg of triamcinolone
	1.5 mg of betamethasone
	1.5 mg of dexamethasone

**Table 3:** Characteristics of commonly used steroids [21–23].

Steroids	Equivalent physiological doses (mg/day)	Anti-inflammatory effect relative to cortisol	Duration of action
Cortisol	20	1	Short acting (8–12 hours)

Prednisone	5	4	Intermediate acting (12–36 hours)
Prednisolone	5	4	
Methylprednisolone	4	5	
Triamcinolone	4	5	

Betamethasone	0.6	25	Long acting (36–72 hours)
Dexamethasone	0.75	30	

**Table 4:** Corticosteroids supplementation protocol in stressful situations.

Stress intensity	Stressful situations	Recommended supplementation
Mild	Minor surgery (e.g., tooth extraction, genioplasty)	25 mg of cortisol on the day of surgery
Moderate	Moderate surgery (e.g., panfacial fracture, two jaw surgery)	50–75 mg of cortisol for 1-2 days
Severe	Major surgery (e.g., head and neck resection and reconstruction)	100–150 mg of cortisol for 2-3 days

## Data Availability

The data supporting this review are from previously reported studies and datasets, which have been cited.
